# Pregnancy trauma and risks of hypoxia, convulsions, cerebral palsy and respiratory complications in offspring

**DOI:** 10.1016/j.jped.2026.101632

**Published:** 2026-10-27

**Authors:** Yao-Chi Hsieh, Ching-Heng Lin, Ming-Chih Lin

**Affiliations:** a Kuang Tien General Hospital, Department of Pediatrics, Taichung, Taiwan; b Taichung Veterans General Hospital, Department of Medical Research, Taichung, Taiwan; c National Yang Ming Chiao Tung University, Institute of Public Health and Community Medicine Research Center, Taipei, Taiwan; d UCL, Department of Epidemiology and Public Health, London, United Kingdom; e National Chung Hsing University, College of Medicine, Department of Post-Baccalaureate Medicine, Taichung, Taiwan; f Taichung Veterans General Hospital, Children’s Medical Center, Division of Neonatology, Taichung, Taiwan; g Chung Shan Medical University, School of Medicine, Taichung, Taiwan

**Keywords:** Trauma, Intrauterine hypoxia, Cerebral palsy, Pregnancy

## Abstract

**Objectives:**

Pregnancy trauma affects 5% to 8% of pregnancies, yet evidence regarding neurological and respiratory consequences for offspring remains limited. The authors investigated the association between pregnancy trauma and adverse neurological and respiratory outcomes in children.

**Method:**

This population-based cohort study used linked data from the Taiwan National Health Insurance Research Database and Maternal and Child Health Database. The study included 2,018,401 mother-infant pairs between 2004 and 2020. Mothers with pregnancy trauma (n = 55,500) were matched 1:4 with mothers without pregnancy trauma (n = 222,000) based on maternal age, neonatal sex, and birth month. Participants were followed up through December 31, 2023. Adjusted hazard ratios (aHRs) were estimated using multivariable Cox proportional hazards regression.

**Results:**

Compared with controls, children of exposed pregnancy trauma had significantly increased risks of intrauterine hypoxia and birth asphyxia (aHR, 1.57; 95% CI, 1.13-2.18), composite neonatal respiratory morbidity (aHR, 1.12; 95% CI, 1.03-1.22), convulsions in newborn (aHR, 1.45; 95% CI, 1.04-2.03) and cerebral palsy, unspecified (aHR, 1.26; 95% CI, 1.06-1.51). Risks were markedly higher when pregnancy trauma required hospitalization (aHR for intrauterine hypoxia and birth asphyxia, 3.29; 95% CI, 1.67-6.50; composite neonatal respiratory morbidity, 1.43; 95% CI, 1.14-1.79) or occurred during the second trimester (hypoxia and asphyxia, 1.88; 95% CI, 1.20-2.93).

**Conclusion:**

Pregnancy trauma is associated with increased risks of adverse neurological and respiratory outcomes in offspring, particularly following severe or second-trimester trauma. These findings highlight the need for pregnancy trauma prevention and underscore the importance of appropriate postnatal care and neurological surveillance for offspring exposed to pregnancy trauma.

## Introduction

Pregnancy trauma, defined in this study as unintentional maternal injury occurring during pregnancy, is a significant clinical concern, affecting an estimated 5–8% of pregnant women [1,2]. Pregnancy trauma encompasses a broad spectrum of injuries of varying severity and mechanisms, including motor vehicle accidents, falls, and other unintentional injuries. Such injuries can have detrimental consequences for both the mother and the fetus [[3], [4], [5], [6], [7], [8]]. Fetal and neonatal complications include preterm birth, respiratory distress syndrome, and perinatal mortality, [4,7,8] while maternal complications encompass antepartum hemorrhage, placental abruption, an increased need for cesarean delivery, and, in severe cases, uterine rupture [3,4].

The leading causes of pregnancy trauma are motor vehicle collisions, falls, and other unintentional injuries [1,2,8,9]. The severity of pregnancy trauma strongly correlates with the risk of adverse maternal and neonatal outcomes [3,4,7]. Specifically, deliveries occurring shortly after the traumatic event are associated with significantly poorer prognoses, [7] including elevated risks of hemorrhage, preterm birth, and perinatal death [7]. Furthermore, women hospitalized for trauma during pregnancy have a higher likelihood of delivering during that admission, which frequently results in unfavorable outcomes [3].

Although pregnancy trauma is a relatively common clinical scenario, research focusing on its long-term neurodevelopmental impact on offspring remains scarce. To date, only a few case series [[10], [11], [12]] and two population-based studies [13,14] have addressed this critical issue.

Therefore, this population-based study aimed to investigate the association between pregnancy trauma and adverse neurological and respiratory outcomes in offspring, including intrauterine hypoxia and birth asphyxia, neonatal convulsions, composite neonatal respiratory morbidity, and cerebral palsy. Cerebral palsy was further evaluated as overall cerebral palsy, unspecified cerebral palsy, and cerebral palsy diagnosed after 3 years of age.

## Methods

### Data sources

The primary data source for this study was the National Health Insurance Research Database (NHIRD). Established in 1995, the NHIRD encompasses nearly the entire Taiwanese population, covering 99.99% of its approximately 23.5 million residents [15,16]. To enable accurate linkage between maternal and offspring records, the authors integrated the NHIRD with the Taiwan Maternal and Child Health Database (MCHD, [17] which is maintained by Taiwan’s Health Promotion Administration (HPA). All data were obtained and analyzed through the Health and Welfare Data Science Center (HWDC). This study was approved by the Institutional Review Board of Taichung Veterans General Hospital (Approval number: CE16270B).

### Study population

Using the linked population-based datasets, the authors identified all live births in Taiwan between 2004 and 2020. To ensure the independence of maternal exposure, only the first live-born child of each mother during the study period was included, resulting in a total of 2,018,401 mother–infant pairs. The authors excluded cases involving suicide or intentional self-harm (n = 7,543) and violence- or assault-related injuries (n = 8,330). The study population was stratified into two groups: mothers who experienced pregnancy trauma (n = 55,500) and those who did not (n = 1,947,028). For the control group, the authors selected cases without pregnancy trauma at a 1:4 ratio, matching for maternal age, neonatal sex, and birth month. This matching process yielded 222,000 individuals in the control cohort (Figure 1). All included offspring were followed for a minimum of 3 years after birth to allow sufficient ascertainment of study outcomes. Accordingly, children born in the final enrollment year (2020) were followed through at least the end of 2023.

**Fig. 1:**
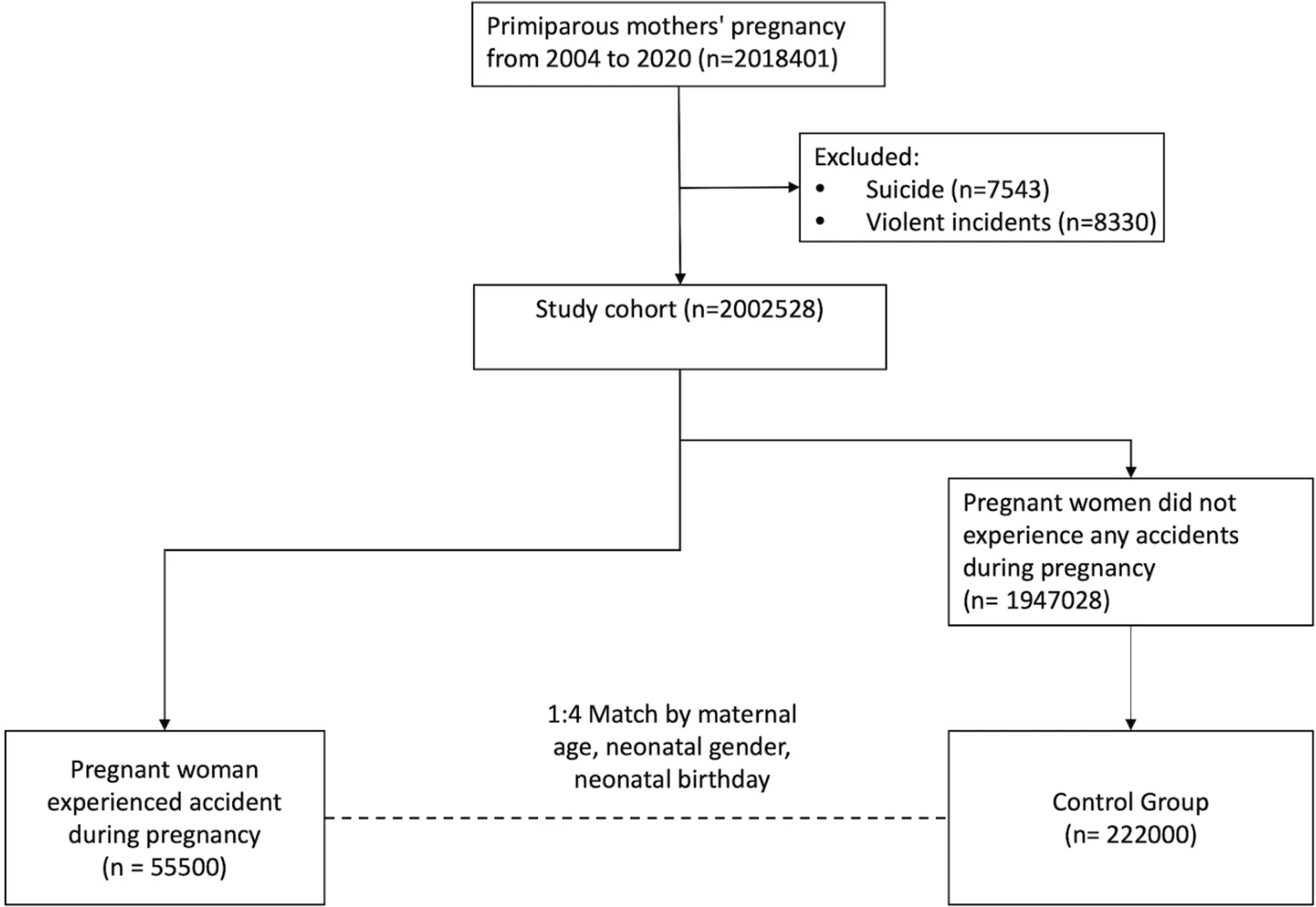
Composition of the study cohort.

### Exposures

Diagnostic data were obtained using the International Classification of Diseases, Ninth Revision, Clinical Modification (ICD-9-CM) and the Tenth Revision (ICD-10-CM). The Taiwan National Health Insurance system utilized ICD-9-CM codes until 2016 and transitioned to ICD-10-CM coding in 2017. Pregnancy trauma was operationally defined as an unintentional maternal injury diagnosed during pregnancy. Pregnancy traumas were identified using ICD-9-CM codes 800–999 and the corresponding ICD-10-CM codes S00–S99 and T00–T88, encompassing a broad spectrum of injuries involving different anatomical regions and mechanisms. To restrict the exposure definition to unintentional pregnancy trauma, intentional injuries were excluded. Specifically, suicidal and intentional self-harm events were excluded using ICD-9-CM codes E950–E959 and ICD-10-CM codes X71–X83, T14.91, while violence- or assault-related injuries were excluded using ICD-9-CM codes E960–E969 and ICD-10-CM codes T74. For the primary analysis, all remaining eligible unintentional injuries were grouped together and analyzed as pregnancy trauma, irrespective of the specific anatomical site or mechanism of injury. Mothers with at least one eligible injury diagnosis during pregnancy were classified as the exposed (pregnancy trauma) group, whereas those without an eligible injury diagnosis were classified as the non-exposed (no pregnancy trauma) group.

### Outcomes

The infant outcomes of interest included intrauterine hypoxia and birth asphyxia (ICD-9-CM 768; ICD-10-CM P20–P21), convulsions in the newborn (ICD-9-CM 779.0; ICD-10-CM P90). Cerebral palsy was assessed using three outcome definitions. Overall cerebral palsy was identified using ICD-9-CM codes 343.x and ICD-10-CM codes G80.x, whereas unspecified cerebral palsy was specifically identified using ICD-9-CM code 343.9 and ICD-10-CM code G80.9. To reduce potential misclassification associated with diagnoses made at younger ages, the authors additionally evaluated cerebral palsy among children diagnosed after 3 years of age. Additional neonatal respiratory outcomes were examined, including respiratory distress syndrome (ICD-9-CM 769; ICD-10-CM P22) and other respiratory conditions of the fetus and newborn (ICD-9-CM 770; ICD-10-CM P23–P28). Because these two respiratory outcomes can be difficult to clearly distinguish in routine clinical practice and administrative databases, they were combined into a composite outcome termed composite neonatal respiratory morbidity to represent the overall burden of neonatal respiratory complications. All participants were followed longitudinally until December 31, 2023.

### Cofactors

Maternal comorbidities, including diabetes mellitus (ICD-9-CM 250.x; ICD-10-CM E10–E14) and hypertension (ICD-9-CM 401–405; ICD-10-CM I10–I15), were ascertained from diagnoses recorded before pregnancy, whereas pregnancy-related complications, including gestational diabetes mellitus (ICD-9-CM 648.0, 648.8; ICD-10-CM O24) and pre-eclampsia and eclampsia (ICD-9-CM 642.4, 642.5, 642.6; ICD-10-CM O11, O14, O15), were identified throughout the entire pregnancy. Multivariable Cox proportional hazards regression models were adjusted for maternal age, maternal comorbidities, pregnancy-related complications, mode of delivery, neonatal sex, multiplicity of birth, birth weight, and gestational age. The same set of covariates was applied consistently across the primary and subgroup analyses to evaluate the association between pregnancy trauma and offspring outcomes.

### Statistical analysis

Demographic characteristics were summarized as frequencies and percentages, and categorical variables were compared using the Chi-square test. Kaplan–Meier survival analysis was employed to estimate the cumulative incidences of intrauterine hypoxia and birth asphyxia, neonatal convulsions, and cerebral palsy. To adjust for potential confounding factors, multivariable Cox proportional hazards regression models were utilized. Subgroup analyses were conducted to examine whether varying severities and circumstances of pregnancy trauma influenced neonatal outcomes. Specifically, the authors assessed the impact of the total number of pregnancy traumas, the interval between pregnancy trauma and delivery, the diagnostic setting in which the injury was identified (emergency department versus inpatient hospitalization), and the timing of the injury (by trimester) on the risk of adverse offspring outcomes. A two-sided p-value of < 0.05 was considered statistically significant for all analyses. Statistical analyses were performed using SAS software (Version 9.4, SAS Institute Inc., Cary, NC, USA).

## Results

### Baseline characteristics

Compared with the control group (n = 222,000), the pregnancy trauma group (n = 55,500) had a higher prevalence of baseline comorbidities, including diabetes mellitus (1.0% vs. 0.7%; *p* < 0.001) and hypertension (1.3% vs. 0.8%; *p* < 0.001). Regarding the mode of delivery, the rate of cesarean section was significantly higher in the pregnancy trauma group (35.1% vs. 33.1%; *p* < 0.001), whereas the incidence of multiple births was lower (1.4% vs. 1.8%; *p* < 0.001). Furthermore, infants born to mothers in the pregnancy trauma group had higher proportions of low birth weight (< 2,500 g; 8.7% vs. 8.0%; *p* < 0.001) and preterm birth (< 37 weeks of gestation; 8.4% vs. 7.9%; *p* = 0.002). These baseline characteristics are summarized in Table 1.

**Table 1:** Comparison of maternal and neonatal demographic characteristics between the pregnancy trauma group and the non-exposed group.

**Characteristics**	**Non-exposed** **(no pregnancy trauma) group** **n = 222,000 (%)**	**Exposed** **(pregnancy trauma) group** **n = 55,500 (%)**	***p*-value**
**Maternal age**			1.00
< 25	58836 (26.5)	14709 (26.5)	
25-29	71192 (32.1)	17798 (32.1)	
30-34	61844 (27.9)	15461 (27.9)	
**≥** 35	30128 (13.6)	7532 (13.6)	
**Maternal comorbidity**			
Diabetes mellitus	1506 (0.7)	530 (1.0)	<0.001
Hypertension	1771 (0.8)	694 (1.3)	<0.001
**Pregnancy-related complication**			
Gestational diabetes mellitus	3041 (1.4)	784 (1.4)	0.44
Preeclampsia	915 (0.4)	241 (0.4)	0.47
**Mode of delivery**			<0.001
Vaginal delivery	148524 (66.9)	36039 (64.9)	
Cesarean section	73476 (33.1)	19461 (35.1)	
**Neonatal Gender**			1.00
Female	106780 (48.1)	26695 (48.1)	
Male	115220 (51.9)	28805 (51.9)	
**Number of babies**			<0.001
Singleton	218103 (98.2)	54737 (98.6)	
Multiple	3897 (1.8)	763 (1.4)	
**Birth weight (grams)**			<0.001
≥2500	204157 (92.0)	50697 (91.3)	
1500-2500	16267 (7.3)	4412 (7.9)	
<1500	977 (0.4)	258 (0.5)	
<1000	599 (0.3)	133 (0.2)	
**Gestational age**			0.002
≥37wks	204356 (92.1)	50863 (91.6)	
34-36+6wks	14346 (6.5)	3797 (6.8)	
28-33+6wks	2747 (1.2)	723 (1.3)	
< 28wks	551 (0.2)	117 (0.2)	
**Number of days from the pregnancy trauma to delivery**			
> 7 days		54200 (97.7)	
≤ 7 days		1300 (2.3)	
**Number of days from the pregnancy trauma to delivery**			
> 14 days		53089 (95.7)	
≤ 14days		2411 (4.3)	
**Total number of pregnancy trauma during pregnancy**			
1		52028 (93.7)	
≥ 2		3472 (6.3)	
**Intrauterine hypoxia and birth asphyxia**	125 (0.1)	49 (0.1)	0.007
**Convulsions in newborn**	127 (0.1)	46 (0.1)	0.03
**Respiratory distress syndrome**	1372 (0.6)	377 (0.7)	0.10
**Other respiratory conditions of fetus and newborn**	1546 (0.7)	432 (0.8)	0.07
**Composite neonatal respiratory morbidity**	2392 (1.1)	669 (1.2)	0.01
**Cerebral palsy**	654 (0.3)	189 (0.3)	0.08
**Cerebral palsy, unspecified**	505(0.2)	159 (0.3)	0.01
**Cerebral palsy (> 3 gt; 3 years old)**	551(0.2)	161 (0.3)	0.08

### Association between pregnancy trauma and adverse neonatal outcomes

Pregnancy trauma comprised a broad spectrum of eligible unintentional maternal injuries identified using ICD-9-CM codes 800–999 and ICD-10-CM codes S00–S99 and T00–T88, after excluding intentional self-harm and violence-related injuries as defined in the methodology section. All eligible unintentional injuries were combined and analyzed as a single exposure category, irrespective of injury type.

Regarding unadjusted clinical outcomes, the pregnancy trauma group demonstrated significantly worse neonatal neurological and respiratory morbidities compared with the control group. Specifically, there were higher incidences of intrauterine hypoxia and birth asphyxia (0.09% vs. 0.06%; *p* = 0.007), neonatal convulsions (0.08% vs. 0.06%; *p* = 0.030), and cerebral palsy, unspecified (0.3% vs. 0.2%; *p* = 0.01). Similarly, the pregnancy trauma group demonstrated a significantly higher incidence of composite neonatal respiratory morbidity (1.2% vs. 1.1%; *p* = 0.010). These unadjusted outcomes are also presented in Table 1.

Using multivariable Cox proportional hazards regression models, the authors adjusted for potential confounders, including maternal age, comorbidities, pregnancy-related complications, mode of delivery, neonatal sex, multiplicity of birth, birth weight, and gestational age. Mothers who experienced pregnancy trauma exhibited significantly elevated adjusted hazard ratios (aHRs) for intrauterine hypoxia and birth asphyxia (aHR 1.57; 95% CI, 1.13–2.18; *p* = 0.008), composite neonatal respiratory morbidity (aHR 1.12; 95% CI, 1.03–1.22; *p* = 0.010), and neonatal convulsions (aHR 1.45; 95% CI, 1.04–2.03; *p* = 0.031). Regarding cerebral palsy outcomes, pregnancy trauma was not significantly associated with the overall risk of cerebral palsy (aHR, 1.16; 95% CI, 0.98–1.36; *p* = 0.08). However, a significant association was observed for unspecified cerebral palsy (aHR, 1.26; 95% CI, 1.06–1.51; *p* = 0.011). When the analysis was restricted to children diagnosed with cerebral palsy after 3 years of age, the association remained non-significant (aHR, 1.15; 95% CI, 0.96–1.37; *p* = 0.13). Overall, these findings demonstrate a significant independent association between pregnancy trauma and an increased risk of adverse infant respiratory and neurological outcomes (Figure 2).

**Fig. 2:**
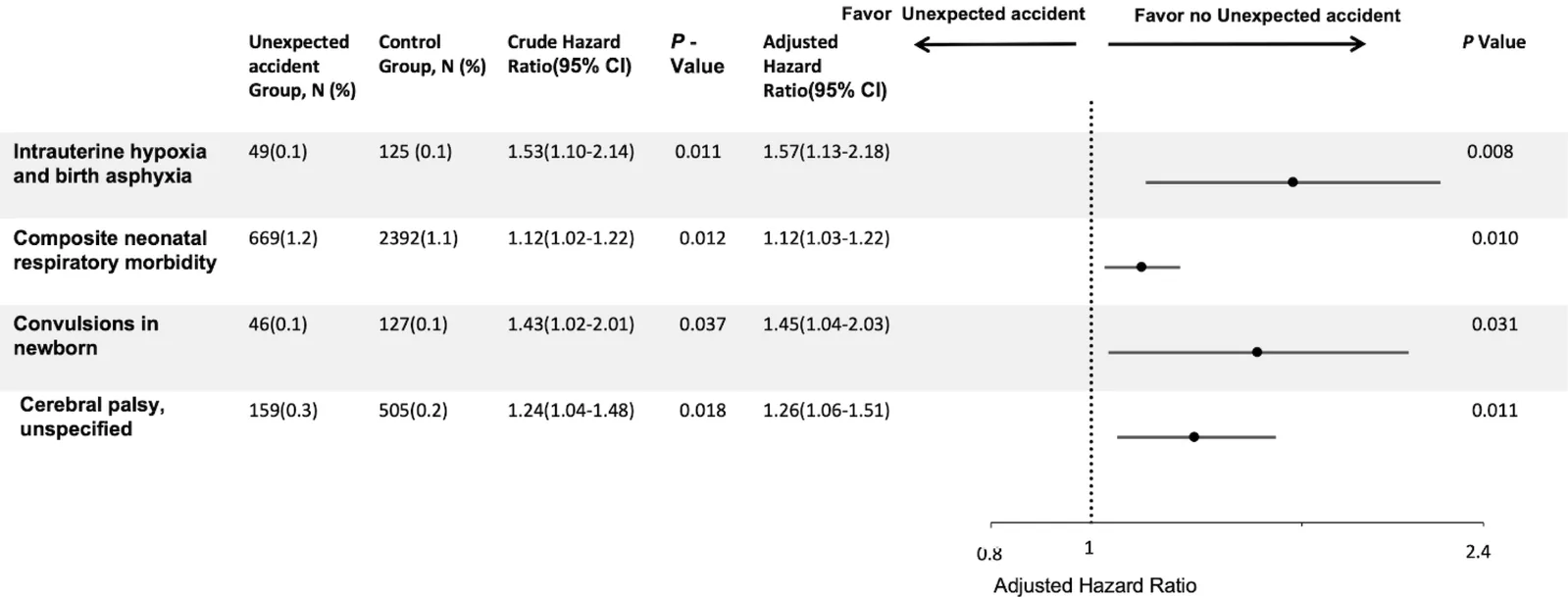
Adjusted hazard ratios for adverse neonatal respiratory and neurological outcomes associated with pregnancy trauma. *CI: confidence interval; Model adjusted for maternal age, maternal comorbidities, pregnancy-related complications, mode of delivery, neonatal sex, multiplicity of birth, birth weight, and gestational age.

### Subgroup analysis of pregnancy trauma severity and timing

The authors further examined whether the severity and timing of pregnancy trauma influenced offspring outcomes. When injury severity was stratified by treatment setting, a gradient effect was observed: compared with the control group, mothers treated in the emergency department had worse infant outcomes, and those requiring inpatient hospitalization exhibited even higher risks. Specifically, pregnancy trauma requiring hospitalization were associated with substantially elevated risks of intrauterine hypoxia and birth asphyxia (aHR 3.29; 95% CI, 1.67–6.50; *p* = 0.001), composite neonatal respiratory morbidity (aHR 1.43; 95% CI, 1.14–1.79; *p* = 0.002), and cerebral palsy, unspecified (aHR 2.10; 95% CI, 1.36–3.26; *p* = 0.001) (Figure 3). Additional subgroup analyses explored injury frequency and timing. Mothers who experienced two or more pregnancy traumas had offspring with a higher likelihood of cerebral palsy, unspecified (aHR 1.62; 95% CI, 0.93–2.81; *p* = 0.09). Regarding the timing of delivery relative to maternal injury, delivery within 7 days after injury was associated with a significantly increased risk of composite neonatal respiratory morbidity (aHR, 1.44; 95% CI, 1.01–2.06; p = 0.044). When a broader 14-day interval was examined, the point estimate remained elevated; however, the association was attenuated and no longer statistically significant (aHR, 1.16; 95% CI, 0.87–1.56; p = 0.31). Notably, pregnancy trauma occurring during the second trimester was associated with a significantly increased risk of intrauterine hypoxia and birth asphyxia (aHR 1.88; 95% CI, 1.20 -2.93; p = 0.006) and cerebral palsy, unspecified (aHR 1.42; 95% CI, 1.10–1.83; *p* = 0.007) in offspring (Table 2).

**Fig. 3:**
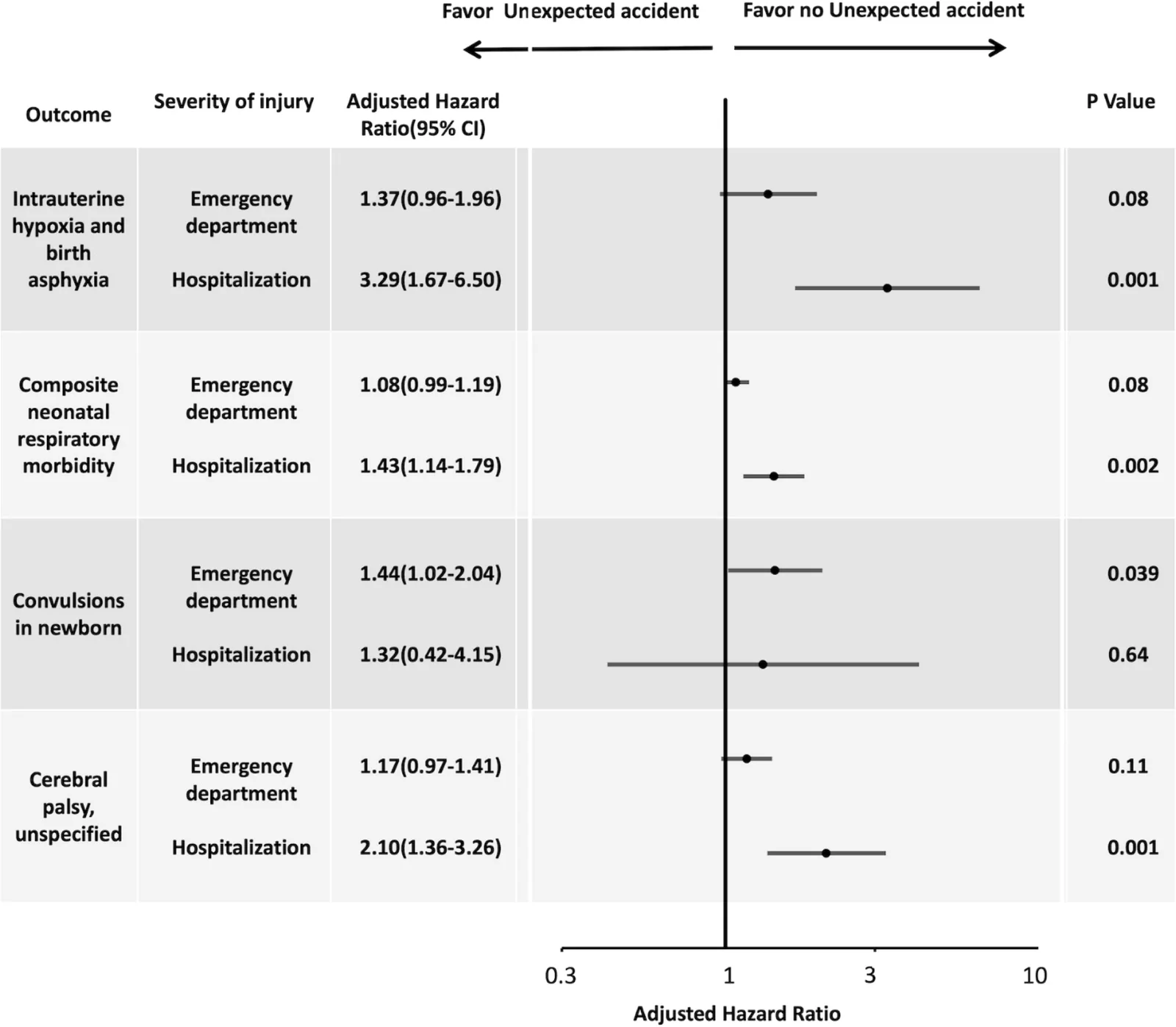
Association between maternal injury severity, defined by treatment setting (emergency department vs hospitalization), and adverse infant respiratory and neurological outcomes. *CI: confidence interval; Model adjusted for maternal age, maternal comorbidities, pregnancy-related complications, mode of delivery, neonatal sex, multiplicity of birth, birth weight, and gestational age.

**Table 2:** Severity of pregnancy trauma and associated infant respiratory and neurological outcomes.

**Characteristics**	**Intrauterine hypoxia and birth asphyxia**					**Composite neonatal respiratory morbidity**					**Convulsions in newborn**					**Cerebral palsy, unspecified**				
	**Case, n (%)**	**aHR**	**95%CI**		***P-value***	**Case, n (%)**	**aHR**	**95%CI**		***P-value***	**Case, n (%)**	**aHR**	**95%CI**		***P-value***	**Case, n (%)**	**aHR**	**95%CI**		***P-value***
**Frequency of injury**																				
Control	125 (0.1)	1.00				2392 (1.1)	1.00				127 (0.1)	1.00				505 (0.2)	1.00			
1	46 (0.1)	1.54	1.10	2.16	0.012	626 (1.2)	1.12	1.02	1.22	0.014	46 (0.1)	1.49	1.06	2.10	0.021	146 (0.3)13 (0.4)	1.22	1.01	1.46	0.038
2 or more	3 (0.1)	1.41	0.45	4.45	0.554	43 (1.2)	1.10	0.81	1.49	0.544		0.50	0.07	3.57	0.489		1.62	0.93	2.81	0.087
**Number of days from the accident to delivery**																				
Control	125 (0.1)	1.00				2392 (1.1)	1.00				127 (0.1)	1.00				505 (0.2)	1.00			
> 7 days	49 (0.1)	1.59	1.14	2.21	0.006	638 (1.2)	1.10	1.01	1.21	0.027	46 (0.1)	1.47	1.05	2.06	0.025	154 (0.3)	1.24	1.04	1.49	0.020
≤ 7 days	0 (0.0)	-				31 (2.4)	1.44	1.01	2.06	0.044	0 (0.0)	-				5 (0.4)	1.26	0.52	3.04	0.613
**Number of days from the accident to delivery**																				
Control	125 (0.1)	1.00				2392 (1.1)	1.00				127 (0.1)	1.00				505 (0.2)	1.00			
> 14 days	49 (0.1)	1.60	1.15	2.24	0.006	623 (1.2)	1.11	1.02	1.22	0.018	46 (0.1)	1.51	1.08	2.11	0.017	152 (0.3)	1.26	1.05	1.51	0.014
≤ 14 days		0.50	0.07	3.60	0.493	46 (1.9)	1.16	0.87	1.56	0.309	0 (0.0)	-				7 (0.3)	0.97	0.46	2.06	0.945
**Timing of injury**																				
Control	125 (0.1)	1.00				2392 (1.1)	1.00				127 (0.1)	1.00				505 (0.2)	1.00			
Trimester 1	21 (0.1)	1.47	0.93	2.34	0.102	316 (1.4)	1.08	0.96	1.21	0.216	21 (0.1)	1.53	0.96	2.43	0.073	65 (0.3)	1.11	0.86	1.44	0.425
Trimester 2	23 (0.1)	1.88	1.20	2.93	0.006	259 (1.3)	1.14	1.00	1.29	0.053	15 (0.1)	1.26	0.74	2.14	0.405	68 (0.3)	1.42	1.10	1.83	0.007
Trimester 3	5 (0.0)	0.92	0.38	2.26	0.857	94 (0.8)	1.21	0.98	1.49	0.074	10 (0.1)	1.55	0.82	2.96	0.181	26 (0.2)	1.20	0.81	1.78	0.372

*Abbreviation: HR, hazard ratio.

*Timing of injury was calculated based on the gestational age at the time of injury. Women exposed to more than 1 injury were classified according to the gestational age at the first injury.

## Discussion

In this large-scale nationwide cohort study of 2,018,401 live births from 2004 to 2020, the authors demonstrated that pregnancy trauma is associated with an increased risk of adverse infant outcomes, including intrauterine hypoxia, birth asphyxia, convulsions in newborns, and composite neonatal respiratory morbidity. Furthermore, our subgroup analyses revealed that greater pregnancy trauma severity, indicated by multiple injury events, injuries requiring hospitalization, and trauma occurring during the second trimester, was associated with poorer neurologic outcomes.

Only a limited number of studies have examined the long-term neurodevelopmental outcomes of children born to mothers who had pregnancy trauma. The earliest report, published by Viljoen in 1995, described a child with multiple neurodevelopmental and structural abnormalities following a maternal road traffic accident early in pregnancy [10]. In 2006, Leroy-Malherbe et al. reported a case series of 18 children who exhibited various severe neurological handicaps following pregnancy trauma during pregnancy, challenging the traditional assumption that fetal outcomes are benign when delivery appears uncomplicated [11]. Similarly, Hayes et al. described 10 children diagnosed with cerebral palsy following pregnancy trauma [12].

To date, only two population-based studies have explored this association. Redelmeier et al. analyzed more than 1.3 million births in Canada, finding that maternal motor vehicle crashes were associated with an increased risk of cerebral palsy among preterm infants [13]. More recently, Ahmed et al. examined over 2.1 million births and demonstrated that in utero exposure to pregnancy trauma was associated with a higher risk of cerebral palsy [14]. Consistent with these findings, our study corroborates a significant association between pregnancy trauma and an increased risk of poor neurologic outcome in offspring.

Because maternal age was an important matching variable, the authors performed additional age-stratified analyses (Supplementary Material Table 1). Pregnancy trauma was associated with unspecified cerebral palsy among mothers aged ≥ 35 years (cHR, 1.72; 95% CI, 1.11–2.66) and with neonatal convulsions (cHR, 1.56; 95% CI, 1.01–2.39; p = 0.043) and composite neonatal respiratory morbidity (cHR, 1.16; 95% CI, 1.03–1.30; *p* = 0.013), among mothers aged 25–34 years. No significant associations were observed among mothers aged < 25 lt; 25 years. Given the small number of events in some strata, these findings should be interpreted cautiously.

The authors used a broad ICD-based definition of pregnancy trauma because restricting the exposure to specific injury mechanisms would substantially reduce the number of eligible cases. In addition, multiple injury diagnoses may occur during the same encounter, and specific injury types may not be consistently captured in administrative claims. Although this approach provides broader capture of pregnancy trauma, potential heterogeneity and exposure misclassification should be considered.

The authors evaluated pregnancy trauma severity using surrogate indicators based on treatment settings. Compared to the control group, mothers requiring an emergency department visit post-injury had worse infant outcomes, while those requiring hospitalization exhibited even higher risks. Specifically, pregnancy trauma necessitating hospitalization was associated with markedly higher risks of intrauterine hypoxia, birth asphyxia, composite neonatal respiratory morbidity, and cerebral palsy in offspring. These findings align with previous studies indicating that maternal hospitalization post-injury is a strong marker of trauma severity and a predictor of poor neurological outcomes in children [3,9,14,18]. The authors also observed a trend where mothers experiencing two or more injury events during pregnancy had offspring with a higher likelihood of cerebral palsy. Although this association did not reach statistical significance, the trend is consistent with prior research, suggesting that a greater frequency of pregnancy trauma may increase the risk of cerebral palsy [14,19].

Furthermore, our findings indicate that pregnancy trauma occurring during the second trimester was associated with the poorest neurological outcomes in offspring, including significantly increased risks of intrauterine hypoxia, birth asphyxia, and cerebral palsy. This pattern contrasts with earlier studies that suggested a greater risk of cerebral palsy following pregnancy trauma in the first trimester [14]. The underlying mechanisms responsible for this heightened vulnerability during the second trimester remain unclear and warrant further investigation. Pregnancy-related complications were included in the adjusted models because of their strong associations with adverse outcomes. However, some complications may occur after pregnancy trauma and potentially act as mediators. Therefore, the adjusted estimates should be interpreted as conditional associations rather than total causal effects.

Unspecified cerebral palsy was selected as the primary outcome because it accounted for most cerebral palsy cases in our cohort (505 of 654 cases, 77.2%). Given the clinical heterogeneity of cerebral palsy and potential limitations of subtype classification in administrative data, the authors additionally examined overall cerebral palsy and cerebral palsy diagnosed after 3 years of age. Neither showed a significant association; therefore, our findings should not be generalized to all cerebral palsy subtypes.

A matched cohort design was selected for this study for several reasons. First, pregnancy trauma was relatively uncommon in the source population, with 55,500 exposed pregnancies compared with 1,947,028 unexposed pregnancies, resulting in a highly imbalanced exposure-group size. Second, the neurological outcomes and composite neonatal respiratory morbidity evaluated in this study were relatively uncommon. Third, matching was performed a priori on maternal age, neonatal sex, and birth month, which were considered important factors potentially associated with pregnancy and neonatal outcomes. Finally, the 1:4 matching ratio was selected because increasing the number of controls beyond approximately four per exposed subject generally provides diminishing gains in statistical efficiency. This cohort study used group matching rather than individual matched-set matching. The non-exposed cohort was selected at a 1:4 ratio according to maternal age, neonatal sex, and birth month, without constructing individual matched sets. Therefore, predefined matched-set strata required for a conditional Cox analysis were not available. The authors instead used multivariable Cox regression adjusting for the matching variables and additionally performed maternal age-stratified analyses as a sensitivity analysis.

### Limitations

This study has several limitations. First, although the NHIRD provides nationwide coverage, it lacks detailed clinical information on injury severity, limiting validation of our indirect severity indicators. Second, reliance on ICD codes may introduce misclassification, particularly for neurological outcomes without standardized clinical assessments. Third, severe trauma resulting in maternal death or immediate pregnancy loss may not have been captured, potentially underestimating the observed risks. Finally, despite adjustment for relevant maternal, pregnancy, and perinatal factors, residual confounding from unmeasured variables cannot be excluded. In addition, the authors did not evaluate the temporal distribution of multiple injury events across pregnancy. Pregnancy trauma also included heterogeneous injury types, which were analyzed as a single exposure category. Therefore, potential differences according to specific injury types or mechanisms could not be assessed and warrant further investigation.

## Conclusion

Pregnancy trauma is associated with increased risks of adverse neurological and respiratory outcomes in offspring, particularly following severe or second-trimester trauma. These findings highlight the need for pregnancy trauma prevention and underscore the importance of appropriate postnatal care and neurological surveillance for offspring exposed to pregnancy trauma.

## Financial sources

This study was supported by Taichung Veterans General Hospital research grants: TCVGH-1144401C, TCVGH-1154401C.

## Role of the funder/sponsor

The funder had no role in the design and conduct of the study; collection, management, analysis, and interpretation of the data.

## Data availability

The data used in this study are not publicly available because of patient confidentiality and data protection requirements. All analyses were conducted through the Health and Welfare Data Science Center (HWDC), Taiwan, using secure remote access to the servers of the Ministry of Health and Welfare. Investigators are required to conduct analyses within this secure environment, and individual-level data cannot be exported or publicly shared. Dr. Ching-Heng Lin (epid@vghtc.gov.tw) had full access to all study data and takes responsibility for the integrity of the data and the accuracy of the data analysis.

## Conflicts of interest

The authors declare no conflicts of interest.

## Appendix Supplementary materials

Image, application 1
