# Supplementary material for: Pregnancy trauma and risks of hypoxia, convulsions, cerebral palsy and respiratory complications in offspring
Source: J Pediatr (Rio J). 2026 Oct 27;103(1):101632. doi: 10.1016/j.jped.2026.101632 (PMC13734835; doi:10.1016/j.jped.2026.101632)
Supplement: Supplementary file 1 [file mmc1.pdf]

Appendix Table 1. Association Between Maternal Injury During Pregnancy and Adverse Infant Respiratory and Neurological Outcomes, Stratified by Maternal Age

Variables	Maternal Age <25				Maternal Age 25-34				Maternal Age ≥35			
	cHR	95%CI		*P-value*	cHR	95%CI		*P-value*	cHR	95%CI		*P-value*
Intrauterine hypoxia and birth asphyxia	1.68	0.89	3.16	0.110	1.43	0.90	2.28	0.135	1.64	0.81	3.33	0.171
composite neonatal respiratory morbidity	1.01	0.84	1.21	0.917	1.16	1.03	1.30	0.013	1.14	0.94	1.38	0.185
Convulsions in newborn	1.63	0.80	3.29	0.178	1.56	1.01	2.39	0.043	0.89	0.37	2.15	0.790
Cerebral palsy	1.04	0.76	1.43	0.805	1.15	0.93	1.42	0.206	1.27	0.84	1.92	0.255
Cerebral palsy, unspecified	1.13	0.80	1.59	0.484	1.17	0.92	1.49	0.196	1.72	1.11	2.66	0.015
Cerebral palsy (>3 years old)	0.95	0.66	1.35	0.754	1.18	0.94	1.49	0.149	1.22	0.77	1.95	0.403
